# Reactivation of Herpes Zoster After Recombinant Vaccine (Shingrix): A Case Report

**DOI:** 10.7759/cureus.34431

**Published:** 2023-01-31

**Authors:** Feras Altukhaim, May Mutlaq, Mohammed Alghamdi, Salha Hakami

**Affiliations:** 1 Dermatology, King Saud University, Riyadh, SAU; 2 College of Medicine, Sulaiman Al Rajhi University, Qassim, SAU; 3 Dermatology, King Salman Hospital, Riyadh, SAU

**Keywords:** varicella-zoster virus, postherpetic neuralgia, shingrix, recombinant vaccine, herpes zoster

## Abstract

Herpes zoster (HZ) is a common contagious dermatological condition that results from reactivation of varicella-zoster virus (VZV), which currently could be prevented by vaccination. We describe a rare case of varicella infection reactivation after routine zoster vaccination in an immunocompetent female in her 60s who developed dermatomal pruritic and vesicular rash one week after receiving Shingrix vaccine, along with fever, sweating, headache, and fatigue. The patient was treated as a case of herpes zoster reactivation with a seven days course of acyclovir. She continued to do well on follow-up with no significant complications. Though uncommon, it is important for healthcare providers to recognize this adverse reaction to expedite testing and treatment.

## Introduction

Herpes zoster (HZ), also known as shingles, results from reactivation of varicella-zoster virus (VZV) infection latent in sensory ganglia and is characterized by a painful, unilateral vesicular eruption in a dermatomal distribution [1]. Vaccination decreases the risk of developing herpes zoster and postherpetic neuralgia, which is the commonest complication of HZ defined by pain and burning sensation lasting more than three months from the onset of the illness [2].

There are two effective types of zoster vaccines as follows: (1) recombinant zoster vaccine (Shingrix), a non-live, recombinant subunit adjuvanted vaccine, given intramuscularly in two doses, was initially approved by Food and Drug Administration (FDA) in 2017 for the prevention of shingles for adults aged 50 years and older, who are at increased risk of the disease from being immunodeficient or immunosuppressed due to disease or therapy [3]. (2) Live zoster vaccine (Zostavax or ZVL), a live attenuated vaccine administered subcutaneously as a single dose in adults aged 60 years and older. By July 2020, ZVL is no longer sold in the United States. However, it continues to be used in many other countries, administered as a subcutaneous injection in adults aged 60 years or older. ZVL, unlike Shingrix, is contraindicated in immunocompromised patients [4].

The above-mentioned vaccines provide great effectiveness in reducing the incidence and the debilitating complications of HZ. Shingrix is the most used vaccine in many countries due to its cost-effectiveness [5]. Shingrix has a 91.3-97.2% efficacy in reducing the risk of HZ which has minimal waning of its effect over many years later [3]. In contrast, ZVL has 68.7% efficacy in the first three years which decreases later on [4]. Though both vaccines were shown to be safe in many literatures with only tolerable reactogenicity at site of injection including pain and myalgia, an unusual but important side effect that needs to be considered is the reactivation of HZ after receiving the vaccine. We describe a rare case of varicella infection reactivation after routine zoster vaccination by Shingrix in an immunocompetent host.

## Case presentation

A 60-year-old female, known to have type two diabetes mellitus, hypothyroidism, and depression, presented to our dermatology clinic for evaluation of skin eruption which started one week after receiving the first dose of Shingrix vaccine and continued for four months thereafter. The patient was complaining of severely itchy and painful rash which started over the left side of the abdominal wall and then progressed to involve the left side of the back and left thigh in L4-L5 dermatomes. At the beginning of the rash, she had high-grade fever and sweating for which she used antipyretics. She also complained of headaches and fatigue that were temporarily relieved with over-the-counter (OTC) painkillers. She didn’t have any similar attack before in her life.

Her diabetes mellitus was controlled with oral hypoglycemic medications. She was also on levothyroxine 50 mcg once daily for hypothyroidism and sertraline 50 mg daily for depression. The patient neither recalls any history of chickenpox nor vaccine against chickenpox. She reported a previous infection by tuberculosis when she was 12 years old for which she received a complete course of anti-tuberculosis (TB) drugs.

**Figure 1:**
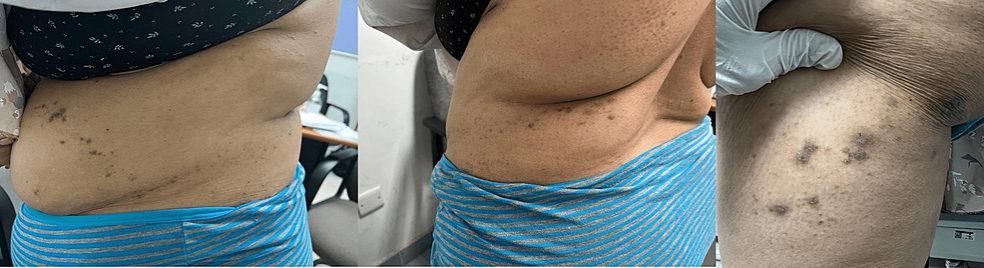
A few scattered vesicular lesions with hyperpigmented macules and patches over the left side of the abdominal wall, back, and left thigh in the L4-L5 dermatomes.

On examination, she was vitally stable. Dermatological assessment showed few scattered vesicular lesions over the left side of the abdominal wall, back, and left thigh in the L4-L5 dermatomes, indicating current active lesions. In addition, she had multiple hyperpigmented macules and patches pointing to previous inflammatory lesions (Figure 1).

Ocular and oropharynx assessments were clear. Her labs showed controlled value of HbA1c of 6.1%, normal liver and kidney function tests, and normal lipid profile. Serology workup was negative for hepatitis B, hepatitis C, and HIV. The patient was diagnosed as a case of herpes zoster based on her typical clinical presentation along with her history of recent vaccination. She was started on acyclovir 800 mg five times a day for seven days. The patient was followed after two and four weeks and she had significant improvement with no active lesions.

## Discussion

Shingrix is a non-live, adjuvanted recombinant vaccine that contains VZV glycoprotein E antigen and adjuvant system AS01B. Glycoprotein E is the most expressed glycoprotein in VSZ-infected cells, it plays a major role in the viral replication and pathogenesis of skin lesions. AS01B is a liposome-based vaccine adjuvant framework that contains two immune stimulants: 3-O-desacyl-4’-monophosphoryl lipid A and saponin QS-21. Monophosphoryl lipid A (MPL) activates innate immunity and results in an increase in cytokine production. QS-21 stimulates CD4+ and CD8+ T cells and promotes antigen-specific antibody responses, thus improving cellular and humoral responses [6,7].

Two large clinical trials (ZOE-50 and ZOE-70) have been done to assess the efficacy of Shingrix in preventing herpes zoster, both demonstrated significant reduction in the risk of reactivation. In adults who were 50 years of age or older, the efficacy of Shingrix against HZ was 97% [8]. The most commonly reported side effects from Shingrix vaccine were pain, redness, and swelling at the sites of injection. Other commonly reported side effects included myalgia, fatigue, headache, shivering fever, and gastrointestinal symptoms [9]. Although clinical trials didn't report herpes zoster reactivation involving the skin as one of the side effects, two case reports mentioned typical skin rash of herpes zoster that occurred a few days after Shingrix vaccine. In one case report, a 73-year-old female presented with a painful itchy skin rash three days after receiving the first dose of Shingrix vaccine which started over the right side of the abdominal wall and then involved her back in the L3-L4 dermatome [10]. The other case report reported a case of a 32-year-old female who developed sharp needle-like pain and itchy rash over her right arm within 24 hours of her first Shingrix vaccine, which continued to involve the upper back, upper chest, right side of midline, involving C4-C8 dermatomes [11].

While there are well-established theories behind herpes zoster reactivation following live attenuated vaccine, it is still unclear how Shingrix can cause reactivation. The authors of a case report about reactivation of herpes zoster keratitis following Shingrix vaccine suggested that the adjuvants of the vaccine may cause an autoinflammatory response, leading to the reactivation. General upregulated immune response to the vaccine was another hypothesis generated by the same authors [12].

Several risk factors may have contributed to HZ reactivation in our case. It is well known that patients older than 50 years of age are at increased risk of reactivation, which was attributed to a decrease in cell-mediated immunity (CMI), an important factor in defense mechanisms against infections [13]. Similarly, major depression is also associated with a decrease in CMI specifically in VZV [14]. Females were also at increased risk for HZ reactivation with no clear evidence behind this gender difference [15]. In addition to old age, depression, female gender, and diabetics were also proven to be associated with increased risk secondary to attenuation in several immunological defense mechanisms including CMI, and innate and adaptive immunity [16].

## Conclusions

In conclusion, reactivation of HZ after the recombinant vaccine in immunocompetent receipt is an unusual event that was reported in only a few patients. This case reports the occurrence of such an incident in an immunocompetent patient. We highlight the need of being vigilant with reactivation of the infection, especially in high-risk candidates. However, this should not conflict with the importance of Shingrix vaccine and its approved benefit in decreasing the incidence and complication of herpes zoster.
